# Single-Photon Detectors for Satellite and CubeSat Quantum Key Distribution: A Systematic Evidence Map

**DOI:** 10.3390/e28030295

**Published:** 2026-03-05

**Authors:** Georgi Tsochev, Elitsa Gieva, Maria Nenova

**Affiliations:** 1 Department of Information Technologies in Industry, Faculty of Computer Systems and Technology, Technical University of Sofia, 1000 Sofia, Bulgaria; 2 Department of Microelectronics, Faculty of Electronic Engineering and Technologies, Technical University of Sofia, 1000 Sofia, Bulgaria; gieva@tu-sofia.bg; 3 Department of Computer Networks, Faculty of Telecommunications, Technical University of Sofia, 1000 Sofia, Bulgaria; mvn@tu-sofia.bg

**Keywords:** single-photon detectors, satellite, QKD, CubeSat, quantum communication, SNSPD, SPAD, avalanche photodiodes, radiation effects, timing jitter

## Abstract

Advancing satellite and CubeSat quantum key distribution (QKD) requires receiver-level engineering trade studies, because secure-key feasibility in space is limited by single-photon detectors (SPDs) operating under SWaP, thermal, and radiation constraints. However, the question arises: does the literature provide sufficiently consistent evidence to guide detector selection for space QKD? This systematic evidence map examines how recent research connects SNSPDs, Si SPAD/APD, InGaAs SPAD/APD, and NFAD variants to CubeSat QKD and space-based quantum communication links. To do so, a concept-token methodology identifies mission contexts and detector families through targeted keywords and key phrases, followed by structured extraction of detection efficiency *η*, dark count rate (DCR), timing jitter, receiver timing window Δ*t*, operating mode, temperature/cooling, and radiation evidence. The results show an upward trend in publications, with many appearing in the last two years. SNSPDs and APD/SPAD families are most regularly discussed, yet key parameters—especially *η*, jitter, and explicit Δ*t*—are reported unevenly, limiting cross-study comparability. CubeSat-tagged studies emphasize APD/SPAD feasibility and radiation-driven DCR evolution, while SNSPDs remain performance-leading but cryogenics-limited. Standardized reporting of *η*, DCR, jitter, Δ*t*, temperature, and radiation conditions emerges as a practical avenue for accelerating deployable space-QKD receivers.

## 1. Introduction

Space-based quantum key distribution (QKD) and closely related quantum communication links are widely regarded as promising enablers of global-scale quantum-secured networks, as they can overcome the distance limitations imposed by fiber attenuation and trusted-node architectures in terrestrial deployments [1,2]. In fiber-based QKD systems, channel loss and noise constrain both achievable distance and key rate, even when advanced protocol variants and decoy-state techniques are employed [3,4]. By contrast, free-space optical links between satellites, airborne platforms, and ground stations can exploit propagation above most of the atmosphere to reduce overall loss and enable intercontinental key exchange, at the cost of stricter requirements on pointing, tracking, and background-light rejection [1,2]. Against this backdrop, an increasing number of demonstrator missions and design studies investigate satellite and CubeSat architectures as practical pathways toward extending quantum-secured connectivity to a global scale [1,2].

In such space-based implementations, the receiver subsystem critically determines whether secret-key generation is feasible under realistic operating conditions. Link loss, atmospheric turbulence, pointing stability, and celestial or anthropogenic background light jointly define the photon budget at the receiver aperture; the single-photon detector (SPD) decides which fraction of those photons is converted into sifted key bits and how much noise is introduced in the process [1,5]. Detection efficiency directly influences the sifted-key rate, whereas dark count rate (DCR), timing jitter, and the effective receiver timing window shape the quantum bit error rate (QBER) and, therefore, the secure-key fraction that can be distilled. As a result, SPD selection is not a marginal component choice but a central design decision in space-QKD receivers, tightly coupled to protocol design, link budget, and on-board processing capabilities [1,5,6].

The key system-level decision in satellite QKD is detector placement. In many downlink demonstrations and designs, the single-photon detectors are located at the ground receiver, which relaxes SWaP and cryogenic constraints but shifts the dominant challenges toward atmospheric channel effects and background rejection at the ground terminal. Conversely, spaceborne detectors become necessary in uplink receiver concepts, inter-satellite links, and other space-to-space quantum communication scenarios, where the detector subsystem must satisfy radiation tolerance, thermal and power budgets, and platform qualification constraints. Because these placement choices fundamentally change which constraints are binding, this review explicitly distinguishes ground-based receiver detectors from on-board (spaceborne) detector subsystems throughout the coding and synthesis.

Unlike terrestrial QKD receivers, satellite and CubeSat implementations must satisfy mission-level constraints that can be as decisive as intrinsic detector performance. These constraints include exposure to ionizing radiation and displacement damage (driving DCR growth and lifetime effects), thermal architecture and cooling feasibility, SWaP (size, weight, and power) budgets—especially for CubeSats and other small platforms—and component space-qualification considerations [1,2]. Consequently, there is no universally optimal detector technology. Superconducting nanowire single-photon detectors (SNSPDs) offer state-of-the-art detection efficiencies, ultra-low noise, and picosecond-scale timing jitter, but their cryogenic cooling requirements impose significant SWaP and integration burdens on the spacecraft [5]. In contrast, avalanche photodiode (APD) and single-photon avalanche diode (SPAD) families, including negative-feedback avalanche diodes (NFADs), are more straightforward to integrate with compact thermoelectric cooling and can leverage mature semiconductor fabrication, yet they typically exhibit higher DCRs, afterpulsing, and pronounced radiation-induced degradation in space environments [5,6]. The choice among these detector families thus involves a multi-dimensional trade-off between performance, reliability, and resource consumption.

A broad literature already documents fiber-based and free-space QKD implementations, as well as reviews of satellite QKD architectures and general SPD technologies [1,2,3,4,5,6]. However, these works typically treat detectors as one element within a larger system; as a result, they do not provide a detector-centric, PRISMA-aligned synthesis that systematically extracts and compares key SPD metrics. For a clear benchmark like that, you need to know detection efficiency, DCR, timing jitter, receiver timing window, operating temperature, and radiation response—in the specific context of satellite and CubeSat QKD. The present systematic review addresses this gap by focusing explicitly on SPD technologies deployed or proposed for space-based QKD and related quantum communication links. Using a concept-token methodology and structured data extraction, the review (i) classifies detector families and mission contexts, (ii) aggregates detector/receiver parameters with a clear distinction between measured and assumed values, and (iii) evaluates engineering suitability for CubeSat-class platforms in terms of SWaP, cooling burden, and radiation sensitivity. In doing so, it aims to provide a coherent evidence base to support detector selection and to motivate more standardized reporting of SPD performance in future space-QKD research.

Concretely, the evidence map contributes: (i) a PRISMA-aligned, detector-centric corpus for satellite and CubeSat quantum links; (ii) structured extraction of η, DCR, timing jitter, and explicit timing windows Δt with explicit tagging of measured versus assumed values; (iii) space-specific synthesis of radiation and SWaP/cooling evidence with reported and transparent marking of reporting gaps; (iv) a CubeSat-oriented suitability matrix separating intrinsic detector performance from subsystem-level integration burden; and (v) a constructive minimum reporting checklist to improve cross-study comparability in future space-QKD detector and receiver publications.

In line with systematic mapping/scoping-style reviews, the aim of this work is to map and structure the evidence base on satellite/CubeSat QKD receiver detector technologies and to characterize engineering-relevant reporting completeness (η, DCR, timing jitter, explicit timing window Δt, operating temperature/cooling, and radiation evidence). We do not seek to estimate pooled effects or answer a narrowly bounded efficacy question; accordingly, meta-analysis and conventional risk-of-bias tools are not applicable to the present objective.

## 2. Related Work

Several reviews and perspective articles have surveyed satellite and airborne quantum key distribution, but none provide a detector-centric, PRISMA-style synthesis of single-photon detectors for space QKD [1,7,8,9]. Bedington et al. review the overall progress in satellite QKD, including mission architectures, orbits, and enabling technologies, and briefly discuss typical detector choices such as Si/InGaAs SPADs and SNSPDs, yet they do not systematically extract η, DCR, timing jitter, timing windows, or radiation evidence from the underlying literature. Subsequent assessments of satellite QKD performance and feasibility similarly treat detector parameters as assumed inputs in link-budget models or protocol analyses, rather than as the primary subject of a structured evidence review [7,8,9].

In parallel, several broader reviews address either quantum-link platforms or single-photon detection technologies more generally [10,11,12,13]. Airborne QKD overviews summarize detector options for aircraft and other high-altitude platforms, providing valuable qualitative insight into environmental and SWaP constraints, but they do not specifically target satellite and CubeSat QKD receivers nor implement systematic study selection and coding. General single-photon detector reviews and perspective pieces cover the physics and performance of SPADs, SNSPDs, and related technologies across a wide range of applications, including quantum communication, yet they are not space-mission specific and do not map detector metrics to space radiation, thermal, and platform-class constraints [11,12,13]. Mission-oriented white papers such as QEYSSat 2.0 contain concise technology overviews and high-level detector parameter tables in the context of particular satellite programs, but they are not designed as literature-wide, detector-focused systematic reviews.

To the authors’ knowledge, no prior work combines a concept-token search strategy with PRISMA-aligned study selection to produce detector-oriented evidence tables and a CubeSat suitability matrix that jointly organize η, DCR, timing performance, timing windows, operating temperature, and radiation evidence for SPDs in satellite and CubeSat QKD [1,7,8,9,14]. This positions the present review as a complementary, detector-centric resource alongside existing system-level and technology-overview articles [1,7,8,9,10,11,12,13,14].

## 3. Materials and Methods

### 3.1. Review Design and Research Questions

This study follows a scoping-style systematic review and follows some of the reporting principles of PRISMA 2020: Reproducible database search, prespecified eligibility criteria, transparent study selection accounting, and structured data extraction/coding to (i) map detector families and mission contexts and (ii) assess completeness and provenance (measured vs. assumed) of engineering-relevant reporting. The synthesis is narrative- and table-based; meta-analysis is not intended because the included studies primarily report heterogeneous device/receiver parameters and design assumptions rather than commensurate effect estimates. The review addresses:

- RQ1: Which SPD technologies are demonstrated or assumed in satellite and CubeSat QKD/space-based quantum communication receivers?
- RQ2: What detector/receiver parameters are reported (e.g., η, DCR, timing jitter, timing window, or temperature), and how do they compare across detector families?
- RQ3: Which space constraints (SWaP, thermal architecture, radiation environment/mitigation, or qualification) most strongly influence detector selection?
- RQ4: What evidence supports CubeSat suitability across detector families?

### 3.2. Data Sources and Search Strategy

The research strategy in this systematic evidence map follows a concept-token approach tailored to single-photon detectors (SPDs) in satellite and CubeSat quantum key distribution (QKD). First, three concept blocks were defined: (A) SPD technologies, (B) QKD/quantum communication, and (C) space mission context. Each block was represented by a set of concept tokens (keywords and key phrases, including common abbreviations) such that any occurrence of a token in a publication indicates an occurrence of the corresponding concept. The literature search was executed in Scopus and Web of Science (final update: 25 December 2025) using database-specific Boolean query strings that combine the three blocks, ensuring that retrieved records simultaneously address detector technologies, quantum communication/QKD, and satellite/CubeSat conditions. After exporting results, records were merged and deduplicated using normalized DOI matching (with title matching where DOI was unavailable). The concept-token counts were retained as structured evidence of topical alignment and were used to support systematic mapping (e.g., detector-family cues and mission-class cues). Concept occurrences were treated as searchable strings across the accessible article content (title, abstract, and full text where available), enabling transparent inclusion decisions and allowing the strictness of screening to be adjusted by specifying minimum token co-occurrence thresholds for the core concepts (Table 1).

The official websites for searching (with the advanced search option) were used by applying queries. The research criteria in Scopus were:
TITLE-ABS-KEY(  (  “single photon detector*” OR “single-photon detector*” OR  “single photon avalanche diode*” OR “single-photon avalanche diode*” OR  SPAD* OR NFAD* OR “negative-feedback avalanche diode*” OR  “avalanche photodiode*” OR  “single photon counting” OR “single-photon counting” OR  “single photon counting module*” OR  “superconducting nanowire single-photon detector*” OR SNSPD  )  AND  (  “quantum key distribution” OR QKD OR  “quantum communication” OR  “quantum cryptography” OR  “entanglement distribution” OR “entangled photon*”  )  AND  (  satellite* OR spaceborne OR “space-borne” OR “space-based” OR  CubeSat* OR cubesat* OR nanosat* OR microsatellite* OR  “low Earth orbit” OR LEO OR  “medium Earth orbit” OR MEO OR  “geostationary orbit” OR GEO OR  “deep space”  ))
The research criteria in Web of Science were:TS=(  (  “single photon detector*” OR “single-photon detector*” OR  “single photon avalanche diode*” OR “single-photon avalanche diode*” OR  SPAD* OR NFAD* OR “negative-feedback avalanche diode*” OR  “avalanche photodiode*” OR  “single photon counting” OR “single-photon counting” OR  “single photon counting module*” OR  “superconducting nanowire single-photon detector*” OR SNSPD  )  AND  (  “quantum key distribution” OR QKD OR  “quantum communication” OR  “quantum cryptography” OR  “entanglement distribution” OR “entangled photon*”  )  AND  (  satellite* OR spaceborne OR “space-borne” OR “space-based” OR  CubeSat* OR cubesat* OR nanosat* OR microsatellite* OR  “low Earth orbit” OR LEO OR  “medium Earth orbit” OR MEO OR  “geostationary orbit” OR GEO OR  “deep space”  ))

The Microsoft Excel software (LTSC MSO (16.0.14334.20440) 64-bit) was used to collect the studies in a spreadsheet file that was shared online with the team. Before adding a study to the spreadsheet, its general characteristics were checked.

After the studies were collected in the spreadsheet by the team members, the eligibility phase was started. Occurrences of concept tokens were manually counted. The articles were open-access, so they were downloaded and searched. For each of them, team members entered the concept tokens from the spreadsheet columns into the document search function to record their occurrences. See the Data Availability Statement for a public link to the spreadsheet file for more details.

### 3.3. Eligibility Criteria

The search strategy required manual counting of multiple concept tokens across full-text scientific articles; counting all conceptual tokens was time-consuming. Therefore, only core concept tokens were counted during the screening phase. A minimum number of occurrences of a concept was set as part of the inclusion criteria. Thus, if an article had fewer than the specified minimum number, it was excluded during the screening stage of the study selection process, before full eligibility assessment.

#### 3.3.1. Inclusion Criteria

- Open-access full text available in the compiled dataset.
- Satellite, CubeSat/nanosatellite, or spaceborne context relevant to free-space quantum links.
- Explicit discussion of photon-counting/SPD technology or detector/receiver constraints transferable to QKD design (e.g., timing-window-limited background rejection, DCR-driven QBER effects, and radiation/thermal constraints).

#### 3.3.2. Exclusion Criteria

- Fiber-only studies without a space/satellite/CubeSat context.
- Protocol-only/theoretical studies with no receiver/detector assumptions relevant to engineering trade-offs.
- Non-archival items lacking sufficient technical detail for extraction.

### 3.4. Study Selection and Deduplication

**Figure 1:**
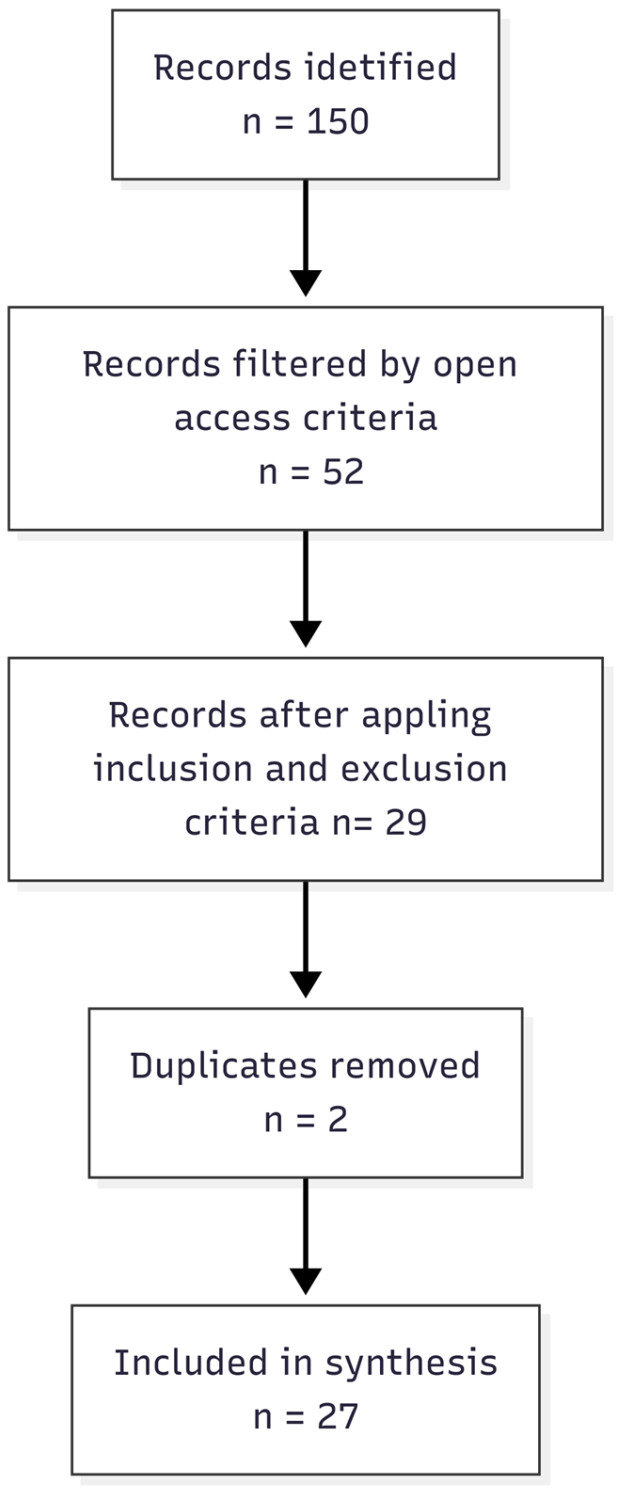
PRISMA-style flow diagram for the compiled open-access dataset.

Records exported from Scopus and Web of Science were merged into a single dataset. Deduplication was performed using normalized DOI matching (including DOI-URL normalization) and title matching when the DOI was unavailable. The compiled dataset contained 29 records; 2 duplicates were removed, and therefore, there were 27 unique included studies in the final dataset (Figure 1).

### 3.5. Data Extraction and Coding

A structured extraction template captured:

- Detector family (SNSPD; InGaAs APD/SPAD; Si APD/SPAD; NFAD; mixed/comparative).
- Detector location (spaceborne/on-board vs. ground-based receiver) and link direction (uplink/downlink/inter-satellite), when extractable from the text/metadata.
- Operating mode (gated/free-running) and receiver timing model elements (explicit timing/gating/coincidence window Δt), where stated.
- Core performance metrics: Detection efficiency η, dark count rate (DCR), and timing jitter.
- Measurement conditions for reported metrics, when stated: Wavelength band, detector temperature, and other relevant operating conditions (e.g., dead time/hold-off and filtering).
- Radiation evidence: (a) Qualitative mention; (b) quantitative irradiation conditions (particle type/energy, fluence/dose) and pre-/post-irradiation DCR where reported; mitigation/annealing/shielding notes, where available.
- SWaP/cooling evidence, when reported: Cooling approach (ambient/TEC/cryogenic), indicative power/mass/volume for the detector subsystem or receiver module, and integration/qualification cues.

To avoid conflating system assumptions with demonstrated detector performance, extracted parameter values were tagged as:

(M) measured/experimental; (A) assumed/model parameter; (R) reported without explicit measurement context; and NR: not reported.

### 3.6. Synthesis Approach and Reporting-Quality Considerations

Due to heterogeneous reporting of detector parameters and test conditions, the evidence map uses narrative synthesis supported by structured tables. Reporting completeness (presence/absence of η, DCR, jitter, Δt, and temperature with contextual conditions) is treated as an engineering analog to risk-of-bias assessment because incomplete parameter reporting can bias technology comparisons and link-feasibility conclusions.

## 4. Results

### 4.1. Included Studies and Corpus Overview

**Figure 2:**
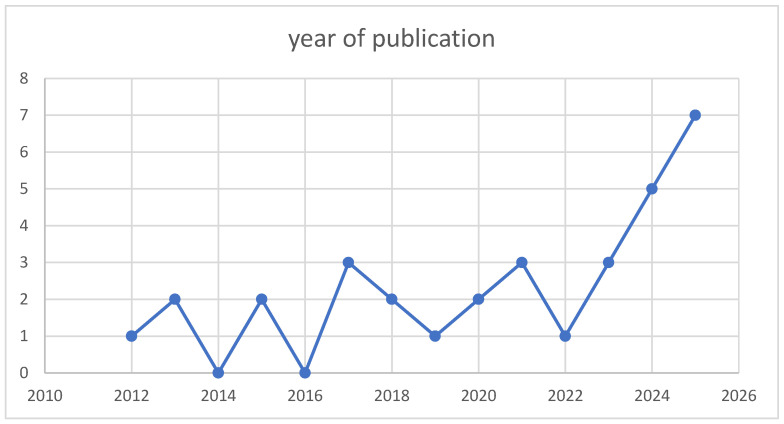
Counts of the researched articles published per year. It is observed that the number of publications has increased in the last two years.

After deduplication, 27 unique open-access studies published between 2012 and 2025 were included. The corpus shows clear acceleration in recent years, with 9/27 studies in 2024–2025 (Figure 2). Platform tagging indicates satellite-class missions/terminals dominate (16/27), while CubeSat/nanosatellite work is substantial (10/27); 1/27 studies do not specify a platform class in a way that can be robustly categorized from metadata and keyword evidence alone. Because detector placement differs across satellite-QKD architectures (ground-based receivers versus spaceborne/on-board receivers), we explicitly code detector location and, where stated, link direction (uplink/downlink/inter-satellite). This structured classification is summarized in Table 2.

### 4.2. Satellite-Class Missions and Terminals

#### 4.2.1. APD/SPAD-Based Satellite Receivers (Si and InGaAs Families; NFAD Subset)

Across satellite-focused studies, APD/SPAD-class detectors appear as a baseline technology because of their relative integration simplicity compared with cryogenic detectors. For satellite QKD/quantum communication receiver concepts, the extracted evidence (Table 3) shows that APD/SPAD implementations are often discussed in the context of timing-window control (sub-ns to ns-class windows when specified) and noise management, where the relevant noise term is the combination of intrinsic dark counts and background-driven accidentals under the adopted timing window Δt (see Discussion, Equations (1) and (2)). Where studies provide quantitative radiation test results, we extract irradiation conditions and the corresponding DCR evolution (Supplementary Material) to support orbit- and lifetime-aware receiver trade studies.

#### 4.2.2. SNSPD-Based Satellite Receivers

SNSPDs are consistently positioned in the satellite-class literature as a high-performance option when ultra-low timing uncertainty and low noise are required (Table 3). Where explicitly reported, SNSPD-focused studies show picosecond-scale jitter and favorable detection-efficiency values, aligning with their role in background-limited optical links, which were tightening the effective timing window, directly reducing accidental detections. However, the dominant satellite engineering constraint for SNSPD adoption is cryogenic cooling, which introduces a payload-level burden (power, thermal interfaces, mechanical stability, and cryocooler integration complexity). Where subsystem-level SWaP/cooling figures are reported, we extract them in Supplementary Material to separate intrinsic detector performance from integration feasibility.

#### 4.2.3. Comparative/Mixed Satellite Studies (System-Level Evidence)

A substantial subset of satellite studies includes comparative or system-level works that reference multiple detector families to quantify mission feasibility under different receiver assumptions. These papers often provide ranges of assumed efficiency and noise rather than a single implemented detector configuration. For this reason, Table 3 tags values as measured (M), assumed (A), or reported without a clear measurement context (R), and Table 5 aggregates performance evidence in grouped form to avoid conflating “single-technology demonstrated performance” with “system-level assumed parameters”.

### 4.3. CubeSat/Nanosatellite Missions

#### 4.3.1. APD/SPAD Detectors for CubeSat Receivers (Baseline Feasibility)

Within the CubeSat/nanosatellite-tagged studies, APD/SPAD-family detectors (Si and InGaAs, including NFAD as an APD-like subset) appear as the dominant baseline option due to their lower SWaP burden and the feasibility of TEC/moderate cooling or near-ambient operation compared with cryogenic systems. Where papers report subsystem-level constraints (cooling approach, indicative power, mass, or volume), we extract them in Supplementary Material; when not reported, entries are explicitly marked NR to make reporting gaps visible. CubeSat receiver concepts repeatedly emphasize background rejection, which is typically addressed via temporal filtering (explicit timing windows when reported) and careful optical filtering and pointing constraints.

Engineering feasibility in CubeSats is strongly shaped by the balance between (i) achievable receiver noise floor and (ii) the spacecraft’s ability to sustain thermal control and radiation mitigation. For APD/SPAD detectors, radiation-induced DCR growth is particularly consequential in small platforms with limited shielding mass, motivating either conservative duty cycles, orbit selection, annealing strategies, or acceptance of reduced mission lifetime/availability.

#### 4.3.2. SNSPDs in CubeSat-Class Systems (Performance-Driven and SWaP-Limited)

SNSPDs appear in CubeSat-tagged studies as a performance-driven alternative when stringent noise and timing constraints dominate the link budget. The extracted CubeSat-tagged evidence shows that SNSPD-related entries offer strong jitter performance and competitive noise behavior, but the limiting factor is typically cryogenic integration. Accordingly, the CubeSat synthesis (Table 4) separates intrinsic detector metrics (η, DCR, jitter, and Δt) from subsystem-level feasibility drivers (cooling and SWaP burden), and measured-only ranges are reported separately from mixed (measured + assumed) ranges wherever the evidence allows. For CubeSat missions, cryogenic cooling imposes a high SWaP cost and complicates thermal design, often pushing SNSPD use toward larger CubeSat form factors and mission profiles that can justify cryogenic payload complexity.

#### 4.3.3. Receiver Timing and Background Rejection in CubeSats

Across CubeSat receiver concepts, explicit or implicit reliance on temporal filtering emerges as a key background-mitigation mechanism. When timing windows are reported, they fall in the sub-ns to ns range, reflecting the need to reduce accidental detections that scale with Δt (Equation (1)).

This makes timing jitter, synchronization architecture, and clock distribution non-trivial co-design elements alongside detector selection, especially for low-photon-flux regimes where background and dark counts can dominate the error budget.

Table 4 summarizes CubeSat-tagged evidence by detector family, including reported ranges of *η*, DCR, timing jitter, and timing window where available, and complements these with qualitative engineering assessments of cooling burden, SWaP/integration complexity, radiation sensitivity, and overall CubeSat suitability.

### 4.4. Cross-Cutting Synthesis: Performance Reporting and Technology Selection

**Figure 3:**
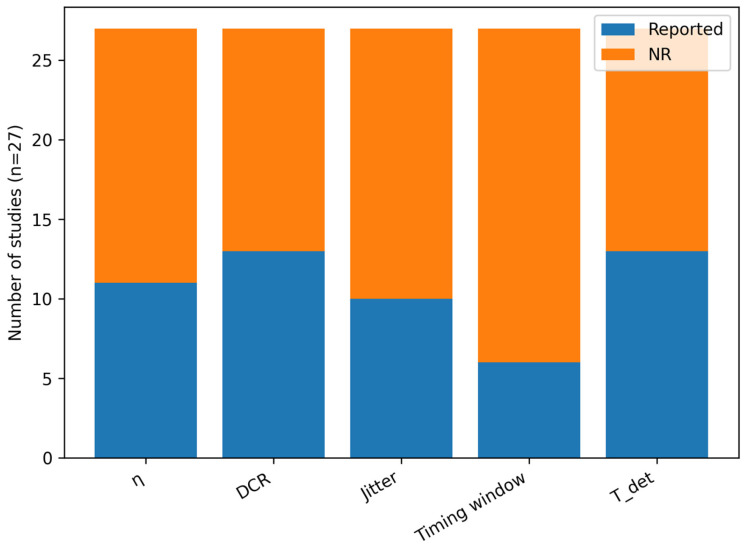
Reporting completeness of key detector/receiver parameters across the included studies (n = 27). “Reported” includes any value tagged as measured (M), assumed (A), or reported without explicit measurement context (R); NR indicates not reported.

**Figure 4:**
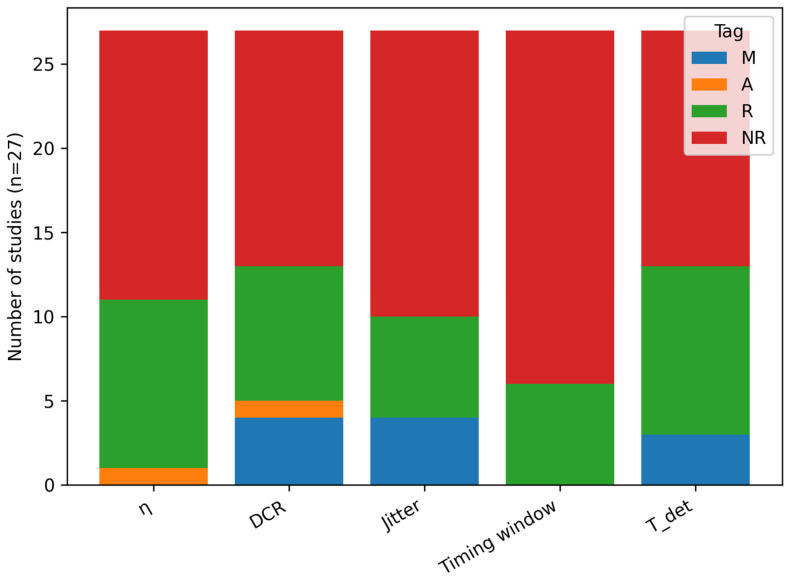
Provenance of extracted parameter values across the corpus (n = 27): measured/experimental (M), assumed/model parameter (A), reported without explicit measurement context (R), and not reported (NR). This separation avoids conflating demonstrated detector performance with modeled feasibility assumptions.

The corpus exhibits non-uniform reporting of detector performance metrics and measurement conditions. While DCR and general timing assumptions appear frequently, η, timing jitter, and—most importantly—the explicit timing window Δt are often missing or reported without standardized conditions (wavelength band, operating mode, detector temperature, dead time/hold-off, and background environment). To make this heterogeneity explicit rather than implicit, we quantify reporting completeness and the provenance of reported values (measured vs. assumed vs. unclear-context) across the included studies (Figure 3 and Figure 4).

This variability justifies the use of narrative synthesis supported by structured evidence tables (Table 3) and grouped summaries (Table 5). It also motivates a reporting-quality checklist as an engineering analog to risk-of-bias assessment: incomplete parameter reporting can materially bias detector comparisons and link-feasibility conclusions because accidental detections and QBER depend jointly on DCR/background and the adopted timing window Δt (Equations (1) and (2)).

**Table 5:** Aggregated summary of reported detector metrics grouped by detector-technology focus.

Group	N Studies	η (n; Range; Median)	DCR (n; Range)	Timing Jitter (n; Range)	Timing Window (n; Range)	T_Det (n; Range)
SNSPD-only	4	4; 30–90%; med 66%	3; 100–100 cps	3; 48–400 ps	1; 3–3 ns	2; 2.1–2.8 K
APD/SPAD-only	9	2; 50–50%; med 50%	5; 20 cps–10 Mcps	1; 600–600 ps	4; 500 ps–12.5 ns	2; 183–225 K
Mixed (SNSPD + APD/SPAD)	11	7; 10–98%; med 63%	8; 25–3 kcps	7; 3 ps–2.6 ns	2; 1–2 ns	7; 2.3–263 K
Unspecified/other	3	0; NR	0; NR	1; 160–160 ps	0; NR	1; 0.1–0.1 K

Groups separate SNSPD-only, APD/SPAD-only, mixed (comparative) studies, and unspecified/other, reporting the number of studies contributing to each metric and the corresponding ranges. This grouping reduces the risk of conflating single-technology demonstrated performance with system-level assumed parameters.

## 5. Discussion

### 5.1. How Detector Metrics Translate to Space-QKD Link Performance

Detector choice impacts space QKD primarily through the interplay of signal detections, accidental detections, and error rates. For a pulsed photon-counting receiver, it is convenient to express noise as the probability of an uncorrelated detection inside the effective timing/gating/coincidence window Δt. If *R_bg_* is the background count rate and *R_d_* is the intrinsic dark count rate (DCR), then the per-symbol noise probability is approximated as:

(1) $$p_{s}\;\;\approx (R_{bg}+R_{d})∆t$$

Let $p_{s}$ denote the per-symbol signal detection probability (set by link loss, optical throughput, and η). Under the standard engineering assumption that (i) optical/visibility imperfections contribute an error fraction $e_{opt}$ on signal detections, and (ii) uncorrelated noise detections contribute a 50% error probability, the *QBER* can be approximated as:

(2) $$QBER\approx \frac{e_{opt}p_{s}+0.5p_{n}}{p_{s}+p_{n}}$$

This decomposition is widely used in satellite-QKD performance analyses to expose how tightening Δt and reducing *R_d_* (and/or background) directly lowers accidental-driven *QBER*, thereby improving the secure-key margin. The approximation assumes that afterpulsing, dead-time effects, and other implementation-specific terms are either negligible, separately modeled, or absorbed into effective rates; these terms should be reported explicitly when relevant.

A generic secure-key rate can be expressed in the usual form:

(3) $$R_{sec}\approx \;R_{sift}[1-f_{EC}H_{2}\left(QBER\right)-H_{2}\left(e_{PH}\right)]\;$$

where $R_{sift}$ is the sifted key rate, $f_{EC}$ is the error-correction inefficiency, *H*_2_(·) is the binary entropy, and $e_{PH}$ is a protocol-dependent phase error term (or equivalent bound).

Together, Equations (1)–(3) clarify why timing jitter and explicit timing windows matter: Reducing Δt and limiting DCR/background directly reduces accidental-driven QBER and increases feasible secure-key rate under realistic link margins.

#### Illustrative Sensitivity of *QBER* to Δt and DCR

**Figure 5:**
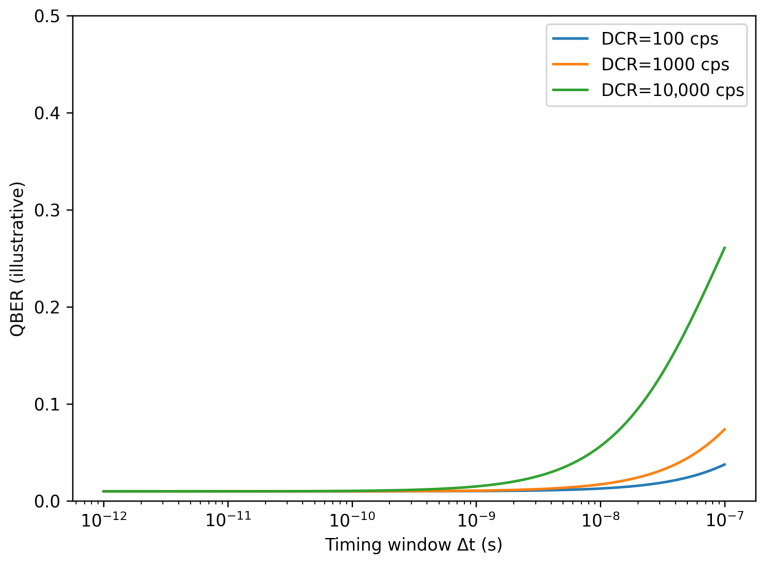
Illustrative sensitivity of QBER to timing window Δt under a simple photon-counting model (Equations (1) and (2)). Curves show QBER versus Δt for three representative DCR levels at fixed background and signal-detection probability; the intent is to visualize how tightening Δt and reducing DCR improve QBER margin.

To illustrate how detector and timing choices map into error budget, Figure 5 plots the *QBER* predicted by Equation (2) as a function of the effective timing window Δt for representative parameters (fixed signal detection probability *p_s_* and optical error eopt) and three dark count levels. The plot is intended as an illustrative sensitivity view rather than a mission-specific prediction: It shows that when *p_n_* becomes comparable to *p_s_*, *QBER* rises rapidly toward the accidental-dominated regime, and that reducing Δt can compensate for higher DCR only up to the point where timing jitter and synchronization constraints limit further window tightening. This makes Δt a system-level co-design variable that links detector physics (jitter, DCR) to receiver architecture (clock distribution, timestamping, and filtering).

### 5.2. Satellite vs. CubeSat: Why “Best Detector” Depends on Platform Class

The Results support a platform-dependent conclusion. Satellite-class payloads can more readily accommodate complex thermal and power subsystems, enabling SNSPD adoption when performance margins justify cryogenics. CubeSats, by contrast, are constrained by SWaP and thermal interfaces; these constraints make APD/SPAD approaches the pragmatic baseline, even when SNSPD performance would be superior in isolation.

### 5.3. SNSPDs: When Cryogenics Is Justified

SNSPDs are most compelling for scenarios where background-limited performance dominates: High-loss links, reduced apertures, partial daylight operations, or aggressive key-rate targets with limited link margin. In these cases, tightening Δt and reducing noise via SNSPD performance can lower QBER enough to justify cryogenic integration. For CubeSats, the decision threshold is often set by cryocooler feasibility and mission power/thermal budgets rather than by detector metrics alone.

### 5.4. APD/SPAD Families: Radiation as the Dominant Reliability Driver

For APD/SPAD detectors, radiation-driven DCR evolution is a dominant concern. DCR is not static in orbit; it evolves with displacement damage, potentially shifting a receiver into an accidental-dominated regime unless mitigation is applied. This makes orbit selection, shielding, annealing strategies, and operating temperature control critical co-design decisions. The literature also reports DCR more consistently than full timing/jitter characterization, even though timing is required to translate DCR into accidental rates via Equation (1).

### 5.5. Recommended Minimum Reporting Set for Space-QKD Detector Papers

A recurring limitation is heterogeneity in reported test conditions and receiver assumptions. Based on the reporting gaps quantified in Figure 3 and Figure 4 and the parameters required to interpret Equations (1) and (2), we propose a minimum reporting checklist for space-QKD detector/receiver studies (Table 6). This checklist ensures that future papers report a complete, comparable set of quantities needed for cross-study synthesis and mission trade studies.

## 6. Limitations

This systematic evidence map is limited by (i) reliance on Scopus and Web of Science exports and (ii) restriction to open-access records in the compiled dataset, which may exclude relevant detector-development work published under subscription access. The included literature also exhibits non-uniform reporting of detector metrics and test conditions; as a result, meta-analysis is not appropriate, and the synthesis is primarily narrative with structured tables. Platform categorization (satellite vs. CubeSat) and orbit cues are derived from study metadata and keyword evidence, which can under-classify studies that omit explicit platform terms. Finally, automated extraction of metrics from PDFs can misinterpret context when authors report values qualitatively or embed parameters in link-budget assumptions; this is partly mitigated by labeling values as measured (M), assumed (A), or reported without explicit context (R), but residual ambiguity may remain for some entries.

## 7. Conclusions

This systematic evidence map synthesized 27 open-access studies (2012–2025) addressing single-photon detectors (SPDs) in satellite and CubeSat quantum key distribution (QKD) and closely related space-based quantum communication links. Across this literature, it becomes clear that detector choice is not simply a matter of maximizing detection efficiency or minimizing dark counts in isolation; instead, it is fundamentally a platform-dependent engineering decision shaped by SWaP, thermal architecture, radiation environment, and receiver timing design. The recent growth in publications—particularly in 2024–2025—reflects accelerating interest in deployable quantum links and, simultaneously, increasing attention to receiver constraints that ultimately determine whether secure-key generation is feasible under realistic mission conditions.

A consistent message emerging from the evidence is that CubeSat-class missions gravitate toward APD/SPAD-family detectors because they are comparatively easier to integrate within tight spacecraft constraints. These detectors are compatible with compact packaging and moderate cooling strategies, enabling practical CubeSat payload designs, but they introduce a dominant reliability challenge: radiation-driven evolution of dark count rate (DCR). In the reviewed corpus, radiation effects are repeatedly treated as a first-order determinant of receiver noise budget and mission lifetime, motivating mitigation through shielding, annealing approaches, conservative duty cycles, and temperature management. At the same time, the literature reinforces that the impact of DCR cannot be evaluated without an explicit receiver timing model, because accidental detections scale with the effective timing window Δ*t* (e.g., Equation (1)). This makes synchronization and temporal filtering inseparable from detector selection, especially for background-limited regimes.

SNSPDs, by contrast, appear repeatedly as the performance-leading detector option, offering excellent timing behavior and attractive noise characteristics that can significantly improve QBER margins in challenging links. However, the principal barrier for SNSPD deployment—particularly on CubeSats—is not detector physics but cryogenic integration and the resulting SWaP and thermal-system burden. As a result, SNSPDs are most compelling when the mission can support cryogenics and when operational conditions (high loss, limited apertures, more aggressive background environments, or higher key-rate targets) make the extra complexity worthwhile. In satellite-class payloads, where power and thermal resources are less constrained than on nanosatellites, SNSPD adoption becomes more realistic and can offer meaningful system-level benefits.

Finally, the evidence map identifies an actionable limitation that directly affects technology selection: inconsistent reporting of detector and receiver parameters across the space-QKD literature. Many studies do not report a complete, comparable set of *η*, DCR, timing jitter, explicit Δ*t*, operating mode/temperature, and radiation test conditions. Improving the standardization of these reported quantities would immediately strengthen cross-study comparability, reduce ambiguity in mission trade studies, and accelerate progress toward robust, deployable satellite and CubeSat QKD receivers.

## Figures and Tables

**Table 1:** The researched concepts and their corresponding concept tokens were searched in the scientific texts. A concept occurrence is viewed as an appearance of strings in the text of the identified studies. These strings are the concept tokens defined as corresponding to the concept.

Concept	Concept Tokens
Single-photon detection ^1^	single-photon detector; single-photon detector; single-photon counting; single-photon counting; single-photon counting module
SNSPD ^2^	superconducting nanowire single-photon detector; SNSPD
SPAD/APD ^2^	single-photon avalanche diode; single-photon avalanche diode; SPAD; avalanche photodiode; single-photon counting
NFAD ^2^	NFAD; negative-feedback avalanche diode
Quantum key distribution ^3^	quantum key distribution; QKD
Quantum communication/cryptography ^3^	quantum communication; quantum cryptography
Entanglement distribution ^3^	entanglement distribution; entangled photon
Space/satellite context ^4^	satellite; spaceborne; space-borne; space-based; CubeSat; cubesat; nanosat; microsatellite; low Earth orbit; LEO; medium Earth orbit; MEO; geostationary orbit; GEO; deep space

^1^ Core SPD concept used to identify photon-counting receiver literature. ^2^ Detector-technology concepts used to classify the SPD family (SNSPD/APD/SPAD/NFAD). ^3^ Quantum-link/application concepts (QKD and closely related space-based quantum communication). ^4^ Space/platform/orbit context concepts (satellite/CubeSat and orbit regimes).

**Table 2:** Included studies and structured classification (application context, platform class, detector location, link direction, orbit keywords, and detector-family cues).

StudyID	Year	Title	Application	Platform *	Orbit *	Detector_KW *
S01	2012	Experimental quantum teleportation over a high-loss free-space channel [15]	QKD + entanglement	Satellite	LEO	APD/SPAD
S02	2013	A comprehensive design and performance analysis of low Earth orbit satellite quantum communication [16]	QKD + entanglement	Satellite	LEO; MEO; GEO	APD/SPAD
S03	2013	Experimental quasi-single-photon transmission from satellite to earth [17]	QKD + entanglement	Satellite	LEO; GEO	Not specified
S04	2015	Large-sensitive-area superconducting nanowire single-photon detector at 850 nm with high detection efficiency [18]	QKD	Satellite	NA	SNSPD; APD/SPAD
S05	2015	Space-Qualified Nanosatellite Electronics Platform for Photon Pair Experiments [19]	Entanglement distribution	CubeSat/nanosatellite	LEO	APD/SPAD
S06	2017	CubeSat quantum communications mission [20]	QKD + entanglement	CubeSat/nanosatellite	LEO; Deep space	APD/SPAD
S07	2017	Laser annealing heals radiation damage in avalanche photodiodes [21]	QKD + entanglement	CubeSat/nanosatellite	LEO	APD/SPAD
S08	2017	Mitigating radiation damage of single photon detectors for space applications [22]	QKD	Satellite	LEO	APD/SPAD
S09	2018	Superconducting nanowire single photon detection system for space applications [23]	QKD	Satellite	LEO; Deep space	SNSPD
S10	2018	Temporal Encoding to Reject Background Signals in a Low Complexity, Photon Counting Optical Communication Link [24]	QKD	CubeSat/nanosatellite	LEO	APD/SPAD
S11	2019	Spaceborne, low-noise, single-photon detection for satellite-based quantum communications [25]	QKD + entanglement	Satellite	LEO; Deep space	SNSPD; APD/SPAD
S12	2020	Registration of the quantum state of a single photon to create a satellite quantum network [26]	QKD + entanglement	CubeSat/nanosatellite	LEO	SPD (generic)
S13	2020	Two-photon quantum interference and entanglement at 2.1 µm [27]	QKD + entanglement	Satellite	NA	SNSPD
S14	2021	QKD terminal for Canada’s Quantum Encryption and Science Satellite (QEYSSat) [28]	QKD + entanglement	Satellite	LEO	APD/SPAD
S15	2021	Radiation-Induced Dark Counts for Silicon Single-Photon Detectors in Space [29]	QKD + entanglement	Satellite	LEO; MEO; GEO	APD/SPAD
S16	2021	Repeated radiation damage and thermal annealing of avalanche photodiodes for space applications [30]	QKD	Satellite	LEO; Deep space	APD/SPAD
S17	2023	Classical clock synchronization for quantum communications using the optical two-way time transfer technique [31]	QKD + entanglement	Satellite	NA	SPD (generic)
S18	2023	Quantum BER estimation modeling and analysis for satellite-based quantum key distribution scenarios [32]	QKD	CubeSat/nanosatellite	LEO	SNSPD; APD/SPAD
S19	2023	Ultrabright polarization-entangled photon pair source for frequency-multiplexed entanglement distribution [33]	QKD + entanglement	Satellite	GEO	SNSPD; APD/SPAD
S20	2024	Impact of visibility limiting conditions on satellite and high-altitude quantum links [34]	QKD + entanglement	CubeSat/nanosatellite	LEO	SNSPD; APD/SPAD
S21	2024	Superconducting single-photon detector with a speed of 5 GHz and a photon detection efficiency of 61% [35]	QKD	Not specified	Deep space	SPD (generic)
S22	2024	THz quantum gap: exploring potential approaches for generating and detecting quantum states [36]	Entanglement distribution	Satellite	NA	SPD (generic)
S23	2025	A new GHz high-speed near-infrared uncooled single photon detector [37]	QKD + entanglement	Satellite	NA	APD/SPAD
S24	2025	QEYSSat 2.0—white paper on satellite-based quantum communication missions in Canada [14]	QKD + entanglement	CubeSat/nanosatellite	LEO; MEO; GEO; Deep space	SNSPD; APD/SPAD
S25	2025	Radiation hardness properties and DCR reduction via laser annealing of SPADs for space applications [38]	QKD	Satellite	LEO	APD/SPAD
S26	2025	Simulation for performance evaluation of satellite-based quantum communication [39]	QKD + entanglement	CubeSat/nanosatellite	LEO; MEO; GEO	SNSPD; APD/SPAD
S27	2025	Advances in Receiver and Detection Systems for Low Earth Orbit Nanosatellite Quantum Communications [40]	QKD + entanglement	CubeSat/nanosatellite	LEO; MEO; GEO; Deep space	SNSPD; APD/SPAD

* Platform/orbit/detector labels in Table 2 are based on systematic keyword evidence and study metadata; mixed labels indicate comparative or system-level papers referencing multiple detector families.

**Table 3:** Evidence Table of extracted detector/receiver metrics across included studies. Values are labeled as (M) measured, (A) assumed/model parameter, and (R) reported without explicit measurement context; NR = not reported. Where stated, measurement conditions (e.g., wavelength band, operating mode, and detector temperature) are recorded; otherwise, they are marked NR.

StudyID	Detector	Mode	η	DCR	Jitter	Timing Window	T_det	Rad
S01	Si	NA	NR	NR	NR	12.5 ns (R)	NR	Quant
S02	InGaAs	NA	50% (R)	1 kcps (R)	NR	0.5 ns (R)	NR	Quant
S03	NS	NA	NR	NR	160 ps (R)	NR	NR	Quant
S04	SNSPD	NA	82% (R)	100 cps (R)	105.3 ps (M)	NR	2.1 K (R)	Quant
S05	Si	NA	NR	NR	NR	4.3 ns (R)	NR	Quant
S06	Si	gated	NR	NR	NR	NR	NR	Quant
S07	NFAD + Si	free-running	NR	NR	NR	NR	NR	Quant
S08	Si	NA	NR	200 cps (M)	600 ps (M)	NR	NR	Quant
S09	SNSPD	NA	50% (R)	100 cps (M)	48 ps (R)	NR	2.8 K (M)	Quant
S10	SNSPD + Si	NA	NR	25 cps (R)	NR	NR	NR	Quant
S11	SNSPD + InGaAs + Si	NA	45% (R)	1 kcps (R)	NR	1 ns (R)	−15 °C (R)	Quant
S12	Si	NA	NR	NR	NR	NR	6 K (R)	Quant
S13	SNSPD	NA	NR	NR	NR	NR	100 mK (R)	Quant
S14	InGaAs + Si	NA	NR	NR	NR	NR	−90 °C (R)	Quant
S15	InGaAs + Si	free-running	NR	NR	NR	NR	−40 °C (R)	Quant
S16	NFAD + InGaAs + Si	free-running; gated	NR	2 kcps (R)	NR	NR	NR	Quant
S17	NS	NA	NR	NR	NR	NR	NR	Quant
S18	SNSPD + Si	NA	30% (R)	100 cps (R)	NR	NR	NR	Quant
S19	SNSPD	NA	90% (R)	NR	0.4 ns (R)	3 ns (R)	NR	Quant
S20	SNSPD + InGaAs + Si	NA	63% (A)	3 kcps (R)	88 ps (R)	2 ns (R)	NR	Quant
S21	SNSPD	gated	90% (R)	NR	80 ps (R)	NR	2.3 K (R)	Quant
S22	NS	NA	NR	NR	NR	NR	100 mK (R)	Quant
S23	InGaAs + SNSPD	free-running; gated	10% (R)	700 cps (R)	350 ps (R)	NR	−35 °C (M)	Quant
S24	InGaAs + Si + SNSPD	free-running; gated	98% (R)	1 kcps (A)	3 ps (M)	NR	1.7 K (R)	Quant
S25	InGaAs + Si	gated	NR	30 kcps→10 Mcps (M)	NR	NR	225 K (M)	Quant
S26	InGaAs + Si + SNSPD	NA	NR	NR	NR	NR	NR	Quant
S27	InGaAs + Si + SNSPD	NA	80% (R)	200 cps (M)	48 ps (M)	NR	10 K (R)	Quant

**Table 4:** CubeSat suitability matrix by detector family (CubeSat-tagged evidence + engineering constraints).

Family	CubeSat_n	η_Range	DCR_Range	Jitter_Range	Window_Range	T_Det_Range	Cool	SWaP	Rad	Suit
SNSPD	7	30–98%	25–3 kcps	3–88 ps	1–2 ns	2.5–258 K	Cryo	High	Med	Med
InGaAs	4	63–98%	200–3 kcps	3–88 ps	2–2 ns	2.5–10 K	TEC/Mod	Med	High	High
Si	9	45–98%	25–3 kcps	3–88 ps	1–4.3 ns	2.5–258 K	Amb/TEC	Low–Med	High	High

**Table 6:** Recommended minimum reporting checklist for space-QKD detector/receiver studies to enable cross-study comparability and link-feasibility interpretation.

Category	Minimum Items to Report	Why It Matters
Detector identification	Detector family/model, operating mode (gated/free-running), wavelength band, and dead time/hold-off	Needed to compare η/DCR/jitter across non-equivalent operating points
Core performance	η, DCR (Rd), timing jitter; state whether values are measured (M) or assumed (A) and under what conditions	Prevents mixing demonstrated performance with modeled feasibility assumptions
Timing model	Explicit timing window Δt (or coincidence window), and how Δt relates to jitter/synchronization	Accidentals scale with Δt (Equation (1)), strongly impacting QBER (Equation (2))
Background environment	Background rate Rbg (or equivalent), filtering assumptions, day/night or illumination regime	Separates detector-limited from background-limited regimes; supports reproducible link analyses
Thermal/cooling	Detector temperature, cooling approach (ambient/TEC/cryo), stability/range	η and DCR are temperature dependent; cooling drives integration feasibility
Radiation context	Orbit assumptions or test conditions (particle type/energy, fluence/dose), shielding, annealing/mitigation, and pre/post DCR, where available	DCR is not static in orbit; radiation drives lifetime and availability
SWaP/integration	Power, mass, volume (detector + cooling + readout), interface constraints, and qualification cues (TRL/space heritage)	Determines feasibility on CubeSats; separates device-level merit from subsystem burden

## Data Availability

The data supporting the reported results can be found at https://tusofiabg-my.sharepoint.com/:x:/g/personal/gtsochev_tu-sofia_bg/IQCnDVt1KUciQIosyiML8Nm6AYAtvb9nrrjr11hamuUjxWM?e=REIA8J (accessed on 26 December 2025).

## Supplementary Materials

The following supporting information can be downloaded at: https://www.mdpi.com/article/10.3390/e28030295/s1; the sample for the bibliometric and content analysis was selected in accordance with the principles recommended for Systematic Literature Reviews—PRISMA [41].

## Abbreviations

The following abbreviations are used in this manuscript:

APD	Avalanche photodiode
DCR	Dark count rate
GEO	Geostationary Earth orbit
LEO	Low Earth orbit
MEO	Medium Earth orbit
NFAD	Negative-feedback avalanche diode
QBER	Quantum bit error rate
QKD	Quantum key distribution
SNSPD	Superconducting nanowire single-photon detector
SPAD	Single-photon avalanche diode
SPD	Single-photon detector
SWaP	Size–weight–power
